# Knowledge regarding breast cancer among Congolese women in Kinshasa, Democratic Republic of the Congo

**DOI:** 10.1002/cnr2.1758

**Published:** 2022-11-20

**Authors:** Stanislas Maseb'a Mwang Sulu, Olivier Mukuku, Arnold Maseb Sul Sulu, Bienvenu Lebwaze Massamba, Désiré Kulimba Mashinda, Antoine Wola Tshimpi

**Affiliations:** ^1^ Department of Oncology Nganda Hospital Center Kinshasa Democratic Republic of the Congo; ^2^ Department of Research Institut Supérieur des Techniques Médicales de Lubumbashi Lubumbashi Democratic Republic of the Congo; ^3^ Department of Pathology Kinshasa University Clinics, University of Kinshsa Kinshasa Democratic Republic of the Congo; ^4^ School of Public Health University of Kinshasa Kinshasa Democratic Republic of the Congo

**Keywords:** breast cancer, breast self‐examination, Kinshasa, knowledge, women

## Abstract

**Introduction:**

Breast cancer is the most frequent type of cancer in women and is characterized by late clinical signs in developing countries, including the Democratic Republic of the Congo. One of the main reasons of death from breast cancer is lack of awareness and screening, which has led to late diagnosis (at an advanced stage). This study aims to measure women's knowledge regarding breast cancer in Kinshasa, in the Democratic Republic of the Congo.

**Materials and Methods:**

A cross‐sectional study of 489 women aged 20–65 years was conducted. Data was collected using a predesigned, tested, self‐administered questionnaire. The questionnaire included specific sections to test the participants' knowledge related to breast cancer and its screening, and practices related to breast self‐examination (BSE). Bivariate and multivariate analyzes were used.

**Results:**

Our results indicated that 22.09% of the participants had good breast cancer knowledge and 77.91% had poor breast cancer knowledge. Overall, 322 (65.85%) participants recognized that BSE is a valuable method for early screening of BSE. In total, 136 (27.81%) respondents had learned to do the BSE and 216 (44.17%) had reported doing it. Two hundred and ninety‐two (59.71%) respondents mentioned that any woman was at risk for breast cancer and 357 (71.78%) mentioned that it was possible to prevent breast cancer. Determinants of an adequate level of knowledge were higher/university educational level (adjusted odds ratio = 2.70; 95% confidence interval: 1.27–5.73; *p* = .010) and having previously been screened for breast cancer (adjusted odds ratio = 2.31; 95% confidence interval: 1.40–3.83; *p* = .001).

**Conclusion:**

The majority (77.91%) of women have demonstrated poor knowledge of signs/symptoms, risk factors, and screening methods of breast cancer. Additional efforts should be made through women's healthcare workers to raise knowledge of breast cancer screening.

## 1 INTRODUCTION

Breast cancer (BC) is the most frequent type of cancer among women in low‐, middle‐, and high‐income regions.^1^ By 2020, according to recent estimates by the World Health Organization (WHO), 2.3 million women with BC and 685 000 deaths from breast cancer had been reported worldwide.^2^ In low‐ and middle‐income nations, BC is a substantial and growing public health problem. According to recent reports, developing countries would account for two‐thirds of new cancer cases by 2035.^3^ Its incidence has increased in many parts of Africa and it is becoming an increasingly urgent concern in developing countries where incidence rates have been observed to grow year after year. This is particularly alarming in view of the scarcity of health promotion, healthcare, and resources contributing to major disparities between developed and developing countries, particularly as most women in developing countries who develop BC seek treatment only when the cancer is at an advanced stage.^1^, ^4^ The breast cancer is a multifactorial disease. The link between breast cancer and a range of risk factors has been indisputably demonstrated. Recent studies demonstrate that risk factors for BC are poorly understood by patients and even by certain healthcare providers,^5^, ^6^ despite the fact that successful cancer prevention necessitates a better understanding of numerous risk components, particularly for proper cancer management. These imminent BC risk factors are demographic factors, personal and family history of BC, nutritional factors, lifestyle, reproductive health factors, and environmental factors.^7^ Breast self‐examination (BSE), clinical breast examination, and ultrasound are only a few of the screening techniques available to find breast cancer at an earlier stage; nonetheless, mammography is still the most common screening tool used globally. Developing countries have limited health‐care resources and a dearth of screening programs. This has led to the reliance of women on BSE as a way to identify breast cancer early and seek early treatment rather than relying on ill‐equipped public health systems in developing African countries.^8^ Mammography screening is only offered at hospitals in RDC and are free for all women. A BSE is the main method in limited resource settings because it is simple, convenient, private, and safe, and it does not necessitate the use of any special equipment.^9^, ^10^ Early diagnosis and prevention of BC involves a a deep knowledge of signs/symptoms, risk factors, and BC detection methods; and it is the obligation of healthcare workers to educate women about these methods.^11^, ^12^ Regrettably, in developing countries, awareness and understanding of the BC's preventative practices among the general population and healthcare workers remains poor, which requires appropriate awareness programs.^13^, ^14^ Several researches have found a poor BC knowledge level among women in developing countries.^5^, ^15^, ^16^, ^17^ However, to the best of our knowledge, no studies on the evaluation of knowledge related BC have been performed in the Democratic Republic of the Congo (DRC) in general, and in Kinshasa in particular. Furthermore, extensive epidemiological researches are required for public health specialists, policy makers, and program managers to address women's healthcare.

The evaluation of BC awareness among women is needed to assess the extent of the problem in the community and to assure the effectiveness of any intervention at the health care system or community level. This study aims to assess women's knowledge regarding BC and its screening in Kinshasa, in the DRC.

## 2 MATERIALS AND METHODS

### 2.1 Study setting and population

This cross‐sectional study was carried out in Kinshasa city, located in the south‐western part of the DRC (longitude 15° 18′ 48″ East and latitude 4° 19′ 39″ South). This city, the capital of the country, extends over 9965 km^2^ with a population estimated in 2021 at 17 million inhabitants. Administratively, Kinshasa city is subdivided into 24 communes and 326 districts.

A two‐stage cluster random sampling was carried out with a minimum sample size of 422 using this formula: *n* = z^2^pq/d^2^, with 95% standard deviation of confidence interval (1.96), prevalence of BC in Kinshasa,^18^ accuracy error at 5% and nonresponse rate of 10%. First, six municipalities in the city were randomly selected from the list of 24 municipalities. Second, in the community setting, women were randomly chosen from the different groups and interviewed. Women of childbearing age, married and unmarried were the eligible population for the study. Severely ill persons, BC patients, hearing impairment, mental disability or inability to respond were exclusion criteria for the study. A total of 489 women aged 20–65 years was included in this study.

### 2.2 Data collection and study variables

The present study was conducted in March 2022. Investigators administered a closed semi‐structured questionnaire based on a literature review and, based on expert opinion, was then pretested with 20 women to validate clarity of meaning and appropriate use of language. The questionnaire used in the present study used validated questions from the study by Heena et al.^17^ The investigators, comprised of the Public Health Students and Nurses, conducted face‐to‐face interviews after receiving guidance on the study protocol and the skills required to administer the questionnaire. The study's purpose was presented to eligible participants, and each participant provided written informed consent for the interview.

Following a thorough review of the literature, numerous survey questions were developed, and the questionnaire was separated into sections. The first section provided demographic characteristics (marital status, age, educational level, religion, personal and family history of BC). The second section included questions related to BC knowledge, which were organized into four headings: potential risk factors, signs/symptoms, and screening methods. Three answers were offered to respondents “Yes”, “No” or “Do not know” to answer this second section. The scale was then dichotomized (every “Correct” response was scored as 1 point, and every “Incorrect” and “Do not Know” response was scored as zero points) and the total BC knowledge score for each respondent was calculated by adding up the points obtained (maximum score of 24). The total score was then classified as poor or insufficient BC knowledge (score of 0 to 14; less than 60% of correct responses) and good BC knowledge (score of 15 to 30; 60% or more of correct responses).

The third section assessed the BSE practice among participants and included specific items about BSE. Respondents were asked if they had heard of the BSE and if they thought the BSE was useful for early detection of BC. Other items in the BSE asked whether they had been taught BSE, whether they practiced BSE, at what age BSE should be practiced, how often BSE should be practiced, when is the best time to do BSE, what action should be taken when an anomaly is found in BSE, and what, in their view, the benefits of BSE.

Internal consistency reliability coefficients (Cronbach's alpha) calculated for the BC knowledge items was .827.

### 2.3 Statistical analysis

Statistical analysis were carried out using STATA version 16 software. Descriptive statistics (frequencies, percentages, means, and standard deviations [*SD*]) were used to describe socio‐demographic characteristics, knowledge related to BC. Student *t*‐test or ANOVA test (when recommended) was used to compare means between different categories of variables.

Bivariate analysis was performed using the Chi‐square test. Then stepwise multiple logistic regression was used to identify factors associated with good BC knowledge. In this case, good BC knowledge was our dependent variable. We included explanatory variables with a bivariate test value of 0.25. *p* < .05 values were considered statistically significant.

## 3 RESULTS

Out of a total of 600 women interviewed, 538 had agreed to voluntarily complete the questionnaire, a response rate of 89.67%. Of these 538 respondents, 489 (90.89%) reported having ever heard of BC and were included in analysis; 49 had never heard of it (9.11%). Main sources of information on BC cited by participants were healthcare providers (40.90%), television/radio (29.65%), friends (23.52%), social media (16.77%), and family members (16.56%).

### 3.1 Participants' socio‐demographic characteristics

The mean age of participants was 29.81 ± 11.90 years. More than half of the participants were single (52.76%). In terms of educational level, 68 (13.91%) respondents were at the primary level, 131 (26.79%) were at the secondary level, and 290 (59.30%) were at the higher/university level. More than half (69.53%) had no occupation. The population surveyed was 37.42% Catholic, 34.36% Protestant/Pentecostal, and 28.22% other religions. Ninety‐three (19.02%) respondents reported having previously been screened for BC. Nine (1.84%) respondents reported having a personal history of BC and 40 (10.1%) reported having a family member or relative who had previously had BC. One hundred and thirty (26.58%) respondents said they had discussed BC or its screening with healthcare professionals in the past 12 months (Table 1).

**TABLE 1:** Socio‐demographic characteristics of the participants with mean knowledge scores

Variable	*N* = 489 *n* (%)	Knowledge score Mean (*SD*)	*p*
*Age*			.0003
20–24 years	196 (40.08)	10.28 (6.43)	
25–29 years	126 (25.77)	10.66 (6.48)	
30–34 years	50 (10.22)	9.12 (5.95)	
35–39 years	26 (5.32)	6.35 (4.11)	
40–44 years	27 (5.52)	7.26 (5.75)	
≥45 years	64 (13.09)	7.63 (5.62)	
*Educational level*			<.0001
Primary	68 (13.91)	7.69 (6.56)	
Secondary	131 (26.79)	7.98 (5.39)	
Higher/University	290 (59.30)	10.67 (6.35)	
*Marital status*			<.0001
Single	258 (52.76)	10.60 (6.46)	
Married	191 (39.06)	8.70 (5.96)	
Widowed/divorced	40 (8.18)	6.67 (5.08)	
*Occupation*			.6915
Employee	149 (30.47)	9.37 (6.17)	
Housewife/Student	340 (69.53)	9.61 (6.33)	
*Religion*			.3236
Catholic	183 (37.42)	10.00 (6.44)	
Protestant	168 (34.36)	8.99 (6.54)	
Others	138 (28.22)	9.59 (5.70)	
*Personal history of BC*			.6632
Yes	9 (1.84)	10.44 (6.08)	
No	480 (98.16)	9.52 (6.29)	
*Have previously screened for BC*			.005
Yes	93 (19.02)	11.81 (6.92)	
No	396 (80.98)	9.00 (6.00)	
*Discussing BC or its screening with healthcare professionals in the past 12 months*			<.0001
Yes	130 (26.58)	11.71 (6.56)	
No	359 (73.42)	8.75 (5.99)	
*Have a family member or relative who has suffered from BC*			.0002
Yes	182 (37.22)	10.89 (6.12)	
No	307 (62.78)	8.74 (6.24)	

Abbreviations: BC, breast cancer; SD, standard deviation.

### 3.2 Respondents' breast cancer knowledge

The breast cancer knowledge score obtained in this study is very low; the mean score was 9.54 ± 6.28 points out of a total of 24 (range: 1 and 24). Therefore, in this study, we considered a score of 0 to 14 to be poor BC knowledge and a score of ≥15 to be good BC knowledge. In dividing participants by knowledge level, 108 (22.09%) had good BC knowledge and 381 (77.91%) had poor BC knowledge. Approximately 20%–36% of participants responded correctly to these potential BC risk factors: alcohol consumption, smoking, high‐fat diet, obesity, early menarche, first child at an advanced age, oral contraceptive use, late menopause, hormone replacement therapy, and lack of physical activity. Only four risk factors (female sex, radiation exposure, family history of BC, and never breastfeeding) were found to be 40% or more correct (Table 2). In the BC signs and symptoms section, 285 (58.28%) participants reported that breast shape change (asymmetry) could be a sign of BC, and 262 (53.58%) participants knew that breast size change could also be a sign of BC.

**TABLE 2:** Participant's knowledge about breast cancer

Items for assessing knowledge about breast cancer	Number (*n* = 489)	Percentage	95% Confidence Interval
*Potential risk factors for developping breast cancer*			
Female sex	324	66.26	61.95–70.31
Radiation exposure	213	43.56	39.23–47.99
Family history of breast cancer	198	40.49	36.23–44.90
Never breast‐feed	195	39.88	35.63–44.28
Advanced age (> 40 years)	177	36.20	32.06–40.55
Hormone replacement therapy	175	35.79	31.66–40.13
Oral contraceptive use	162	33.13	29.10–37.42
High‐fat diet	150	30.67	26.75–34.90
Smoking	147	30.06	26.17–34.27
Obesity	144	29.45	25.58–33.64
Alcohol consumption	139	28.43	24.61–32.58
Physical inactivity	124	25.36	21.70–29.40
First child at late age (>30 years)	116	23.72	20.17–27.69
Late menopause (>55 years)	110	22.49	19.02–26.40
Early onset of menarche (<12 years)	98	20.04	16.73–23.82
*Signs/symptoms which you think are related to breast cancer*			
Change in breast shape (asymmetry)	285	58.28	53.86–62.57
Change in the breast size	262	53.58	49.15–57.95
Abnormal nipple discharge	259	52.97	48.54–57.35
Painful or painless breast mass	257	52.56	48.13–56.94
Lump under armpit	226	46.22	41.84–50.65
Inversion/pulling in of nipple	189	38.65	34.44–43.04
*Methods of diagnosis*			
Self‐breast examination	258	52.76	48.33–57.15
Clinical breast examination by doctor	200	40.9	36.63–45.31
Mammography	256	52.35	47.92–56.74
*Mean (SD) total score for the knowledge scale*	9.54	(6.28)	(extrêmes: 1–24)
*Level of knowledge based on the total score*			
Poor (score of 0–14)	381	77.91	74.03–81.37
Good (score of 15–24)	108	22.09	18.63–25.97

### 3.3 Respondents' breast self‐examination knowledge and practices

Table 4 shows results of participants' BSE knowledge and practice. Overall, 322 (65.85%) respondents agreed that it was a useful tool for early detection of BC. A total of 136 (27.81%) respondents had learned to do BSE and 216 (44.17%) had reported doing it. Overall, 178 (36.40%) participants knew how to use BSE correctly, 152 (31.08%) reported that BSE is practiced weekly; only 84 (17.18%) knew that the best time for BSE is a few days after the first day of the menstrual cycle. For the 273 non‐BSE respondents, reasons were not knowing how to do it (72.16%), not finding it important (14.65%), and not expecting to do BSE (13.19%) (Table 3).

**TABLE 3:** Knowledge and practice of breast self‐examination

Questions/statements for assessing knowledge and practice of breast self‐examination	Number (*n* = 489)	Percentage	95% Confidence Interval
*Breast self‐examination is a useful tool for early detection of breast cancer*			
Yes	322	65.85	61.54–69.91
No	44	9.00	6.77–11.86
Do not know	123	25.15	21.51–29.18
Do you know how to practice breast self‐examination?			
Yes	226	46.22	41.84–50.65
No	182	37.22	33.05–41.59
Do not know	81	16.56	13.53–20.12
I have been taught about			
Yes	136	27.81	24.03–31.94
No	299	61.15	56.75–65.36
Do not know	54	11.04	8.56–14.13
Do you want to know more about breast self‐examination?			
Yes	403	82.41	78.79–85.53
No	34	6.95	5.02–9.56
Do not know	52	10.63	8.20–13.68
Do you practice breast self‐examination regularly?			
Yes	216	44.17	39.83–48.60
No	225	46.01	41.64–50.44
Do not know	48	9.82	7.48–12.77
Breast self‐examination is done by			
The set of all fingers	68	13.91	11.12–17.26
The right hand palpates the left breast using the tips of the first three fingers and the left hand palpates the right breast	178	36.40	32.26–40.76
Palpation with one or two fingers	11	2.25	1.26–3.98
Do not know	232	47.44	43.06–51.87
Time for breast self‐examination			
Weekly	152	31.08	27.14–35.32
Monthly	81	16.56	13.53–20.12
Yearly	28	5.73	3.99–8.15
Do not know	228	46.63	42.25–51.06
The best time to do breast self‐examination			
At the beginning of each month	59	12.07	9.47–15.25
A few days before the menstrual cycle	37	7.57	5.54–10.26
5–7 days after the first day of menstrual flow	84	17.18	14.09–20.77
Do not know	309	63.19	58.83–67.35
Reasons not to practice breast self‐examination^a^			
I do not know how to do it	197	72.16	66.44–77.39
I do not think that's important	40	14.65	10.68–19.41
I do not expect to have breast cancer	36	13.19	9.41–17.79

^a^ *n* = 273.

### 3.4 Determinants of good breast cancer knowledge

Table 4 shows the bivariate analysis between socio‐demographic characteristics of the respondents and good BC knowledge. Significant associations were found between good BC knowledge and the following variables: age, educational level, marital status, previous BC screening, and discussing or screening with healthcare providers in the past 12 months (*p* < .05).

**TABLE 4:** Bivariate analysis of good breast cancer knowledge

Variable	Knowledge		Crude odds ratio [95% Confidence Interval]	*p*
	Good (*n* = 108) *n* (%)	Poor (*n* = 381) *n* (%)		
*Age*				
<40 years	95 (23.87)	303 (76.13)	1.88 [1.00–3.53]	.047
≥40 years	13 (14.29)	78 (85.71)	1.00	
*Educational level*				
Primary	9 (13.24)	59 (86.76)	1.00	
Secondary	15 (11.45)	116 (88.55)	0.84 [0.35–2.05]	.714
Higher/Universitary	84 (28.97)	206 (71.03)	2.67 [1.27–5.63]	.008
*Marital status*				
Single	70 (27.13)	188 (72.87)	4.57 [1.38–23.93]	.005
Married	35 (18.32)	156 (81.68)	2.77 [0.80–14.77]	.105
Widowed/divorced	3 (7.50)	37 (92.50)	1.00	
*Occupation*				
Employee	31 (20.81)	118 (79.19)	1.00	
Housewife/Student	77 (22.65)	263 (77.35)	1.11 [0.70–1.78]	.651
*Religion*				
Catholic	47 (25.68)	136 (74.32)	1.47 [0.88–2.44]	.137
Protestant/Pentecostal	32 (19.05)	136 (80.95)	1.00	
Others	29 (21.01)	109 (78.99)	1.13 [0.64–1.98]	.668
*Personal history of BC*				
Yes	3 (33.33)	6 (66.67)	1.78 [0.28–8.52]	.421
No	105 (21.88)	375 (78.13)	1.00	
*Have previously screened for BC*				
Yes	34 (36.56)	59 (63.44)	2.51 [1.53–4.10]	.0001
No	74 (18.69)	322 (81.31)	1.00	
*Discussing BC or its screening with healthcare professionals in the past 12 months*				
Yes	43 (33.08)	87 (66.92)	2.23 [1.42–3.51]	.0004
No	65 (18.11)	294 (81.89)	1.00	
*Have a family member or relative who has suffered from BC*				
Yes	48 (26.37)	134 (73.63)	1.47 [0.96–2.28]	.078
No	60 (19.54)	247 (80.46)	1.00	

**FIGURE 1:**
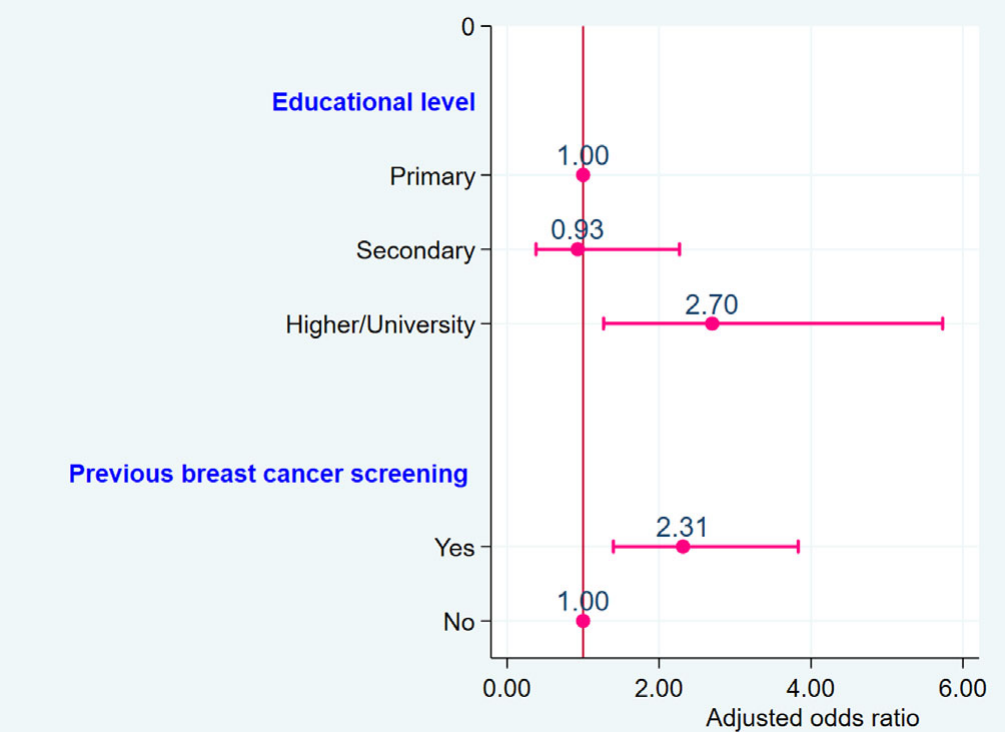
Determinants of good breast cancer knowledge

Multiple logistic regression showed that respondents with higher/university educational level were more likely to have good BC knowledge than those with a primary or secondary educational level (adjusted odds ratio [aOR] = 2.70; 95% Confidence Interval [95% CI]: 1.27 to 5.73; *p* = .010). Similarly, respondents who had previously been screened for BC had significantly good BC knowledge than those who had never been screened (aOR = 2.31; 95% CI: 1.40 to 3.83; *p* = .001) (Figure 1).

## 4 DISCUSSION

As previously stated, BC incidence and death rates are rising in many parts of Africa. BC can be avoided if diagnosed early.^19^ In comparison to developed countries, BC affects African women at a younger age, incurring socioeconomic constraints. Most cases are diagnosed at advanced stages, resulting in higher mortality rates.^1^ The increasing trend in BC incidence in Africa indicates that many young women still do not receive early BC screening.

The breast cancer awareness is vital in disease diagnosis and prevention, and primary healthcare clinics are the health system's first point of contact with women and families.^20^ Increased knowledge of risk factors, symptoms/signs, and screening methods of BC will lead to early BC diagnosis and management and a high survival rate.^21^

The present study showed poor BC knowledge among respondents in general. The results of the survey showed that there is a small proportion of respondents (22.09%) with good knowledge on the set of items evaluated, including knowledge of signs/symptoms, risk factors, and screening methods of BC. Our results seem to be lower compared to a Saudi study which showed that 45.6% of women had good knowledge.^20^ In addition, a study conducted in Monastir (Tunisia) showed that only 8% of respondents had good BC knowledge.^5^ Another study of women in Wardha (India) showed that less than 20% of the participants had good knowledge of BC symptoms and risk factors.^15^ Thus, these results suggest that healthcare providers should be encouraged to incite women to use BC screening methods to reduce the BC incidence by providing accurate information on BC and its screening. More structured educational campaigns with hands‐on sessions are also crucial, as the study demonstrates that there is a significant association between previous BC screening and good BC knowledge (aOR = 2.31; 95% CI: 1.40 to 3.83; *p* = .001).

The present study also found a significant association between higher/university educational level (aOR = 2.70; 95% CI: 1.27 to 5.73; *p* = .010) and good knowledge of BC. These findings may help to explain some of the differences in medical seeking behavior observed in women with BC symptoms in the DRC. Respondents with good BC knowledge were significantly higher/university educational level. In many studies, higher educational level has also been positively associated with BC screening.^22^ Highly educated participants were more likely to obtain information and more receptive to healthcare education.^23^ Previous studies in developed countries have identified low educational level as an indirect measure of lower socioeconomic status that has been linked to be associated with late‐stage BC diagnosis and increased mortality rates.^24^, ^25^

Though that the efficacy of BSE as a screening tool has been debated,^26^, ^27^ it is suggested that women use this method of self‐detection. The present study found that only 27.81% of women reported having been trained to perform BSE and 44.17% of participants had previously practiced BSE. Our results appear to be superior to those reported in previous studies ranging from 0.4% to 38.5%.^20^, ^28^, ^29^, ^30^, ^31^, ^32^, ^33^ The present study found that 72.16% of women argued that lack of BSE knowledge or skills was the main barrier to regular BSE practice. In addition to the lack of information, fear of BC screening results is another barrier to screening among participants. Primary healthcare centers have a key role in raising breast health awareness and prevention actions among women. Therefore, exploring women's BC perceptions, BC screening practices, and factors influencing these practices in the DRC in general, and in Kinshasa in particular is important to encourage this preventive action. For example, primary healthcare services need to provide more health education and strategies for better screening opportunities for BC.

The present study has some limitations. First, its cross‐sectional nature is a limit; it was carried out at a single moment, or over a short period of time. It provides an overview of the results and their related characteristics at that time, and the results could have been different if another time period had been chosen. Second, data were collected in only one province, limiting the generalization of results to all Congolese women. Future researches should cover diverse populations from various provinces to analyze factors associated with BC knowledge and screening methods among Congolese women and provide national recommendations that will influence the entire country. Finally, another potential limitation may be that interviewees may be biased (even inadvertently) in communicating information about themselves that they perceived to be fairly intimate.

## 5 CONCLUSION

The majority of respondents have inadequate awareness of BC signs/symptoms, risk factors, and screening methods. Additional efforts should be made through women's healthcare workers to raise knowledge level of BC screening. This means that we must continue to highlight the importance of primary healthcare for early detection of BC. Therefore, this study calls for advocacy and broader intervention to improve knowledge about BC among women in Kinshasa, with particular reference to poorly educated women.

For health care professionals, who can ultimately play a key role in educating the community about the risk factors for BC and the significance of early detection, it is urgent to develop educational programs based on credible and targeted data.

## AUTHOR CONTRIBUTIONS

**Stanislas Maseb'a Mwang Sulu:** Conceptualization (equal); data curation (equal); investigation (equal); project administration (equal); resources (equal); validation (equal); writing – original draft (equal). **Olivier Mukuku:** Conceptualization (equal); formal analysis (equal); methodology (equal); software (equal); writing – original draft (equal). **Arnold Maseb Sul Sulu:** Data curation (equal); investigation (equal); project administration (equal); resources (equal). **Bienvenu Lebwaze Massamba:** Investigation (equal); supervision (equal); writing – review and editing (equal). **Désiré Kulimba Mashinda:** Formal analysis (equal); methodology (equal); supervision (equal); writing – review and editing (equal). **Antoine Wola Tshimpi:** Conceptualization (equal); methodology (equal); supervision (equal); writing – review and editing (equal).

## CONFLICT OF OF INTEREST

The authors declare that they have no conflicts of interest.

## ETHICS STATEMENT

Informed consent was obtained from every respondent prior to the interviews and no compensation or incentives were provided to respondents for this study.

## Data Availability

Not applicable.
